# Median Nerve Stimulation Based BCI: A New Approach to Detect Intraoperative Awareness During General Anesthesia

**DOI:** 10.3389/fnins.2019.00622

**Published:** 2019-06-19

**Authors:** Sébastien Rimbert, Pierre Riff, Nathalie Gayraud, Denis Schmartz, Laurent Bougrain

**Affiliations:** ^1^Université de Lorraine, CNRS, Inria, LORIA, Nancy, France; ^2^Université Côte d’Azur, Inria, Sophia-Antipolis Méditerrannée, Athena Team, Nice, France; ^3^Le Centre Hospitalier Universitaire (CHU) Brugmann, Université Libre de Bruxelles, Brussels, Belgium

**Keywords:** brain-computer interface, median nerve stimulation, motor imagery, anesthesia, intraoperative awareness

## Abstract

Hundreds of millions of general anesthesia are performed each year on patients all over the world. Among these patients, 0.1–0.2% are victims of Accidental Awareness during General Anesthesia (AAGA), i.e., an unexpected awakening during a surgical procedure under general anesthesia. Although anesthesiologists try to closely monitor patients using various techniques to prevent this terrifying phenomenon, there is currently no efficient solution to accurately detect its occurrence. We propose the conception of an innovative passive brain-computer interface (BCI) based on an intention of movement to prevent AAGA. Indeed, patients typically try to move to alert the medical staff during an AAGA, only to discover that they are unable to. First, we examine the challenges of such a BCI, i.e., the lack of a trigger to facilitate when to look for an intention to move, as well as the necessity for a high classification accuracy. Then, we present a solution that incorporates Median Nerve Stimulation (MNS). We investigate the specific modulations that MNS causes in the motor cortex and confirm that they can be altered by an intention of movement. Finally, we perform experiments on 16 healthy participants to assess whether an MI-based BCI using MNS is able to generate high classification accuracies. Our results show that MNS may provide a foundation for an innovative BCI that would allow the detection of AAGA.

## 1. Introduction

Waking up during a surgery is a haunting experience, both for patients, who consider it as the worst in their lives (Pomfrett, 1999), and for healthcare personnel, who fear this situation (Tasbighou et al., 2018). This phenomenon, called “accidental awareness during general anesthesia” (AAGA), can be defined as an unexpected awakening of the patient during a surgical procedure under general anesthesia (Pandit et al., 2014; Almeida, 2015). This situation occurs when the depth of anesthesia induced by anesthetic concentration is not enough to compensate for surgical and environmental stimuli and prevent awakening (Myles et al., 2004; MacGregor, 2013). Although the statistics are still under debate, the estimated number of AAGA in high-risk practices is up to 1% (Sebel et al., 2004; Avidan et al., 2008; Xu et al., 2009). The percentage of patients affected by AAGA may appear low, but considering the hundreds of millions of general anesthesia performed each year around the world (Weiser et al., 2016), the occurrence of this phenomenon is in fact high. Therefore, new solutions are required to better prevent it (Sebel et al., 2004; Monk and Weldon, 2011).

The main problem for patients experiencing AAGA is the explicit or implicit memory of this distressing experience which can cause severe trauma, termed post-traumatic stress disorder (PTSD) (Osterman et al., 2001). The PTSD following AAGA should not be underestimated: it can last several years and have a severe impact on the victim's life (Avidan and Mashour, 2013; MacGregor, 2013; Almeida, 2015). After experiencing AAGA, more than 70% of patients are reported to be suffering from PTSD (Leslie et al., 2010). They are frequently associated with an increased risk of suicide (Hendin, 1991) and often lead to anxiety, insomnia, flashbacks, chronic fear, avoidance tendencies, loneliness, irritability, concentration difficulty, and lack of confidence in the medical staff (Schwender et al., 1995; Lau et al., 2006; Bischoff and Rundshagen, 2011; MacGregor, 2013; Pandit et al., 2014; Almeida, 2015). AAGA also generates a high anxiety level in anesthesiologists (Xu et al., 2009), and is in the top 3 causes of legal action taken against hospitals (Pandit et al., 2014) which can be expensive if the claim is successful (Mihai et al., 2009).

There are currently two ways to monitor the depth of anesthesia: observing clinical features (e.g., heart rate, blood pressure, movement, sweating; Schafer and Stanski, 2008); or using electroencephalographic (EEG) analysis, mainly of the frontal cortex activity. Unfortunately, an anesthesiologist's observation of clinical signs is not enough to prevent AAGA during surgery (Punjasawadwong et al., 2014). Indeed, observing clinical signs is but an indirect way of monitoring the patients' cerebral state. Hence, it does not always permit the prediction of AAGA before it occurs. New indexes using part of the EEG signal at the frontal level have been employed to prevent AAGA, such as the Bispectral Index (BIS), the Patient State Index (PSI) or the Entropy (Li et al., 2008; Kent and Domino, 2009). Although these devices are already in use (Punjasawadwong et al., 2014; Liang et al., 2015), some studies have failed to demonstrate a superiority of these monitors compared to clinical surveillance or end-tidal anesthetic gas (ETAG) (Avidan et al., 2008; Mashour and Avidan, 2015). Moreover, a number of studies have shown the unreliability of these techniques (Schneider et al., 2004; Schuller et al., 2015). The concentration measurement of anesthetic gases can also be an interesting way to quantify the depth of anesthesia, since it is a measurement and not an estimation, the latter being the case for monitoring anesthesia depth under intravenous products like propofol (Avidan et al., 2008). However, anesthetic gases are much less widespread in Europe (Absalom et al., 2016). In addition, current practices aim to reduce the concentration of anesthetic agents as much as possible in order to reduce post-operative cognitive dysfunction and morbidity (Pandit and Cook, 2013). In fact, most monitoring techniques are less reliable when the concentration of anaesthetic is increased (Mashour et al., 2011) which is why no technique is currently satisfactory and sufficient to evaluate the depth of general anesthesia and detect intraoperative awareness.

**Figure 1:**
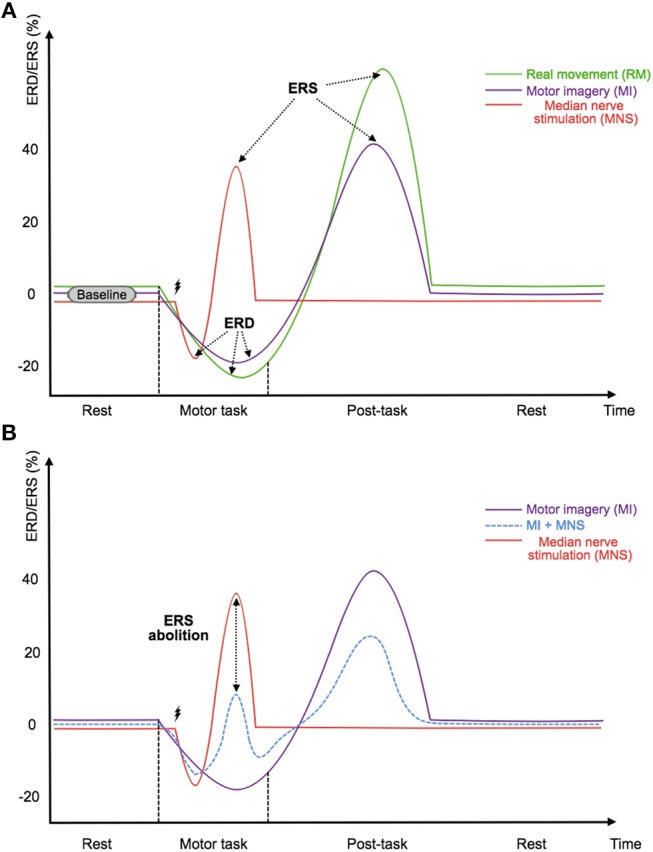
**(A)** Illustration of the timings and amplitudes of the desynchronization and the followed synchronization induced by a real movement, a motor imagery, and a median nerve stimulation according to Salenius et al. (1997),Schnitzler et al. (1997), and Neuper and Pfurtscheller (2001) in the mu and beta frequency bands. **(B)** Illustration of the expected timing and amplitudes of the desynchronization and the followed synchronization induced by a median nerve stimulation during a motor imagery according to Salenius et al. (1997),Schnitzler et al. (1997),Neuper and Pfurtscheller (2001), and Kilavik et al. (2013) in the mu and beta frequency bands. The time scale is not precisely detailed.

Intraoperative awareness leads to this kind of testimony: “*I couldn't breathe*, ***couldn't*** ***move****or open my eyes, or tell the doctor that I wasn't asleep*.” Such testimonies show that, during AAGA, the first reaction from a patient is usually to move to alert the medical staff of this terrifying situation (Ghoneim et al., 2009; Pandit et al., 2014). However, in the majority of surgeries, the patient is curarized, which causes a neuromuscular blockage and inhibits any movement (Tasbighou et al., 2018). Presently, a real movement (RM) or a Motor Imagery (MI) can be detected by analyzing the EEG signal, such as in Brain-Computer Interfaces (BCI, Jonathan Wolpaw, 2012). Detecting RM or MI using EEG is feasible because both the preparation phase and the motor execution phase present power variations in the mu and the beta frequency bands (Pfurtscheller and Lopes da Silva, 1999). These sensorimotor rhythms are characterized, before and during an imagined movement, by a gradual decrease of power in the mu-alpha (7–13 Hz) and beta (15–30 Hz) bands; and after the end of the motor imagery, by an increase of power–mainly–in the beta band. These modulations are respectively known as Event-Related Desynchronization (ERD) and Event-Related Synchronization (ERS) or post-movement beta rebound (Pfurtscheller, 2003; Hashimoto and Ushiba, 2013; Kilavik et al., 2013; Clerc et al., 2016) (Figure 1A).

According to these observations, it would be possible to discover AAGA by detecting an intention of movement from the patient. In 2016, Blockland et al., studied the effect of propofol, a commonly used anesthetic, on the EEG signals of the motor cortex. They verified the relevance of this approach for improving monitoring of AAGA (Blokland et al., 2016). In this study, patients were not completely anesthetized and were asked to perform movements according to sound beeps while an increasing dosage of anesthetic was administered to them. This first approach shows that the BCI domain could contribute to the issue of AAGA. However, the study conducted by Blockland et al. was based on synchronous active communication, i.e., the voluntary subject was explicitly asked to perform a movement during the experiment after a visual and audible signal, which does not realistically reflect the conditions during intraoperative awareness. Therefore, a strategy must be found to design a passive BCI whose task would be to detect the intention of movement of an AAGA victim. In particular, this new BCI should not be based on motor actions previously planned over time by the experimenter and performed by the patient according to specific auditory or visual markers, but rather on the accidental reaction of a patient experiencing AAGA.

The design of such a BCI presents us with two challenges. The first challenge is to be able to detect the intention of movement of a person who is a victim of AAGA without any time markers. This is equivalent to continuously analyzing the EEG signal with few indications regarding the time phases to be studied. While there exist some BCIs that do not use time markers or triggers (known as asynchronous BCIs), the literature clearly shows that their classification rate is lower than that of a synchronous BCI with triggers (Nicolas-Alonso and Gomez-Gil, 2012). The second challenge is therefore to obtain a high level of accuracy, which would guarantee the reliability of the BCI device so that it can be used with patients. The accuracy obtained for a MI vs. Rest classification in the BCI field in general remain low and should be improved to create a reliable device which can be used in hospitals.

To satisfy these two requirements, we propose the use of median nerve stimulation (MNS) and show that it is a very promising approach. Indeed, previous studies have shown that a painless stimulation of the median nerve induces an ERD during the stimulation while an ERS appears after the stimulation (Salenius et al., 1997; Schnitzler et al., 1997; Neuper and Pfurtscheller, 2001) (Figure 1A). More interestingly, a very long motor task performed during a MNS abolishes the patterns previously generated by this stimulation. The gating hypothesis suggests that patterns are contracting (Kilavik et al., 2013) (Figure 1B). If this hypothesis is verified it could make the detection of AAGA with a passive BCI possible. Indeed, we can imagine a routine system where the patient would be stimulated at the median nerve, and the analysis of ERD and ERS modulations of the motor cortex would be used to find out if the patient has an intention to move. Unfortunately, very few studies exist on this topic, and the effect of a MNS during a MI needs to be investigated further, especially for a shorter MI. In addition, no study has shown that a MI + MNS vs. MNS classification results in better accuracies than a MI vs. Rest classification, suggesting that MNS could be used as a trigger.

The objective of this study is to analyze the EEG activity over the motor cortex and (i) verify that median nerve stimulation generates desynchronizations (ERD) and synchronizations (ERS); (ii) confirm that they are modulated by an intention of movement; and (iii) demonstrate that a classification based on this phenomenon would be more effective than conventional classification based on modulations generated by an intention of movement vs. resting state. In order to achieve the above, we recorded 128 EEG signals from 16 voluntary healthy subjects who had performed 3 motor tasks (a real movement, a motor imagery, a MNS during a MI) and reacted to a MNS. To show the influence of a MI on the ERD and ERS generated by a MNS, we computed time-frequency and topographic maps and a classification based on MNS+MI and MNS only. Our results indicate that a MI significantly modulates the ERDs and ERSs generated by a MNS and also that classification based on MNS is more efficient than conventional classification based on MI vs. rest. These results are promising for creating a BCI that detects AAGA.

## 2. Materials and Methods

### 2.1. Participants

Sixteen right-handed healthy volunteers (8 females; 19 to 57 years-old; 28.56 ± 13.3 years old) were recruited for this study. All voluntary subjects satisfied the inclusion criteria (right-handed, between 18 and 60 years-old, without medical history which could have influenced the task, such as diabetes, antidepressant treatment, or neurological disorders). This experiment followed the statements of the WMA declaration of Helsinki on ethical principles for medical research involving human subjects (World Medical Association, 2002). In addition, participants signed an informed consent which was approved by the ethical committee of Inria (COERLE, approval number: 2016-011/01) as it satisfied the ethical rules and principles of the institute.

### 2.2. Experimental Tasks

**Figure 2:**
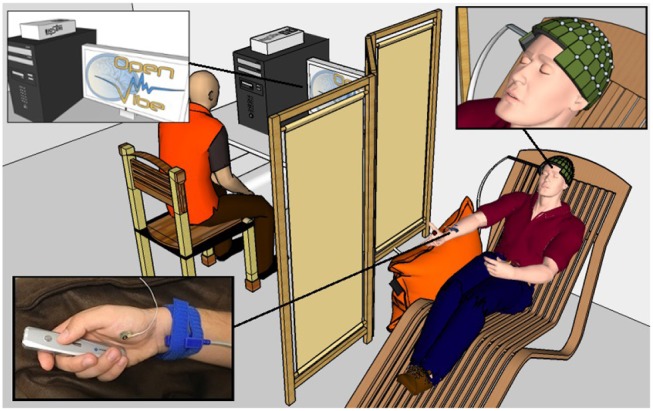
A healthy voluntary subject is lying on a comfortable chair with his eyes closed. His legs rest on a footrest and his right forearm rests on a cushion to prevent movement. The OpenViBE software records 128 EEG electrodes and delivers starting and stopping beeps and stimulations of the median nerve when necessary according to the experimental conditions. The subject physically or mentally presses and releases a remote button. The operator displays the EEG signals during the experiment.

The aim of this research is to investigate the occurrence of motor patterns under 4 different conditions : real movement (RM), motor imagery (MI), median nerve stimulation during a motor imagery (MI + MNS), and median nerve stimulation during rest (MNS) (Figure 2). The first two conditions were designed to assess the reliability of our experimental setup and data processing by comparing these results to the literature. The last two conditions were the core of our study and aim at showing that a MNS can be used and is more helpful as a trigger to improve the detection of intraoperative awareness.

#### 2.2.1. Condition 1: Real Movement

**Figure 3:**
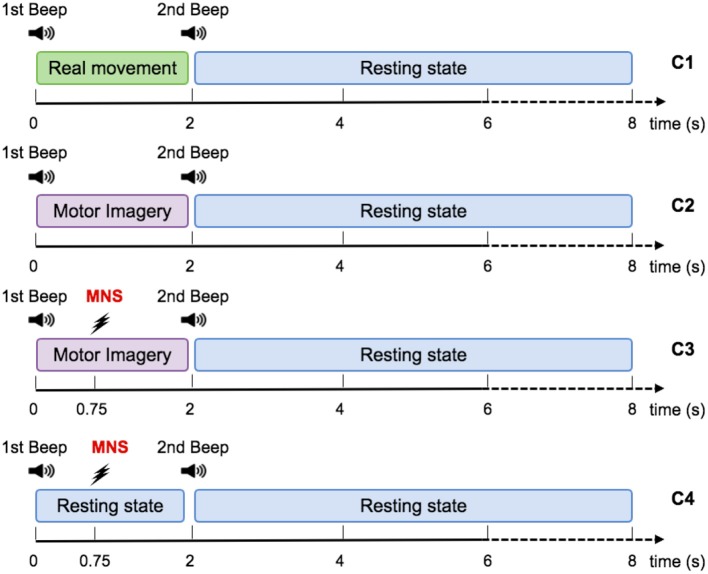
Representation scheme for one trial. Timing schemes of a trial for C1, C2, C3, and C4. For all motor tasks, one low frequency beep indicates when to start the task. For the MNS+MI condition, the MNS occurs at 750 ms after the first beep. The end of the MI is announced by a high frequency beep and followed by a rest period of 6 s.

The RM condition (C1) consisted of an isometric grasp between the thumb and the index finger on a pointer button (Figure 2). A low frequency beep indicated when the subject had to start the movement. The grasping task was maintained during 2 s. Then a second beep indicated when the subject had to stop pressing the pointer button and the task's end (Figure 3). The states of the pointer button were recorded as triggers and allowed us to know exactly when the participant executed and stopped the RM. This simple movement, easy to understand and execute, generates enough brain activity changes which can be observed in EEG (Shibasaki et al., 1993).

#### 2.2.2. Condition 2: Motor Imagery

For the MI condition (C2), subjects had to imagine the previous movement, i.e., they had to try to feel a maximum of sensations caused by the real movement, but without any movement. Similarly to condition 1, a low frequency beep indicated when the subject had to start the motor imagery, the grasping MI was maintained during 2 s, then a second beep indicated the end of the imagined task (Figure 3).

#### Condition 3: Motor Imagery With a Median Nerve Stimulation

During the MI + MNS condition (C3), subjects had to perform a motor imagination while their median nerve was stimulated 750 ms after the start of the motor imaginary task (Figure 3). Uniformly to the previous conditions, a low frequency beep indicated when the subject had to start the motor imagery and a high frequency beep indicated when to stop it. We chose the 750 ms of delay according to the reaction time of the average person, in order to stimulate during the ERD corresponding to the imagination's start. The duration of the stimulation was 100 ms and stimulation intensity was adapted for each subject and varied between 8 and 15 mA.

#### 2.2.3. Condition 4: Median Nerve Stimulation Only

The MNS only condition (C4) consisted of a series of stimulation of the median nerve during rest. We placed the two electrodes of stimulation on the wrist according to the standards (Schnitzler et al., 1997; Kumbhare et al., 2016). We considered as an inclusion criterion for our population of subjects the fact that the stimulation was not felt as painful and that it caused a slight movement between the thumb and the index finger of the voluntary subject. The stimulation intensity was adapted for each subject and varied between 8 and 15 mA.

### 2.3. Experimental Design

Each participant took part in one session of 120 min divided in 4 phases: (1) installation of the EEG cap (40 min); (2) selection of the intensity of median nerve stimulation needed to produce a micro movement between the thumb and index finger (10 min); (3) execution of RM, MI, MI + MNS, and MNS in runs during which participants had to perform the different motor tasks (60 min); (4) uninstallation and debriefing (10 min).

The study contained 4 conditions: real movement (RM), motor imagery (MI), median nerve stimulation during motor imagery (MI + MNS), and stimulation only (MNS). These conditions were completed on the same day and divided into two runs of 26 trials each, representing 52 trials for each condition. The duration of one trial was 8 ± 1 s (Figure 3). The runs were randomized for each subject in order to avoid fatigue, gel drying, or other confounding factors that might have caused possible biases in the results. At the beginning of each run, the subject remained relaxed for 15 s. Breaks of a few minutes were taken between runs to prevent fatigue of the subject.

For the entirety of the experiment, the subject were seated in a comfy chair including a leg rest, with their right arm resting on a pillow, a presentation remote placed in their right hand. The subject didn't actively hold the remote, it was simply resting in their hand with the button placed under their thumb. Subjects were asked to keep their eyes closed (Figure 2).

### 2.4. Data Acquisition

EEG signals were acquired using the *OpenViBE* platform (Renard et al., 2010) with a *Biosemi Active* Two 128-channel EEG system, arranged in the *Biosemi*'s ABC system covering the entire scalp at 2,048 Hz. Among all registered sites, some of the electrodes were localized around the primary motor cortex, the motor cortex, the somatosensory cortex, and the occipital cortex, which allowed us to observe the physiological changes due to the real movement, the kinesthetic motor imagery, and the median nerve stimulation (Salenius et al., 1997; Schnitzler et al., 1997; Guillot et al., 2009; Filgueiras et al., 2017). In the Biosemi^TM^ system the ground electrodes used were two separate electrodes: Common Mode Sense (CMS) active electrode and Driven Right Leg (DRL) passive electrode located over the parietal lobe. Impedance was kept below 10 kΩ for all electrodes to ensure that the background noise in the acquired signal was low. An external electromyogram (EMG) electrode was added in order to verify that there was no movement during the MI task.

### 2.5. Data Pre-Processing

All offline analyses were performed using the EEGLAB toolbox (Delorme and Makeig, 2004) and Matlab2015b (The MathWorks Inc. Natick, MA, USA). The data was processed in General Data Format (GDF). Considering the large number of electrodes used in this study (e.g., =128) and the purpose of this research (motor patterns over the motor cortex) we chose to use a common average referencing (CAR) performed using EEGLAB (Dien, 1998; Lei and Liao, 2017). The results were also visualized by applying a Laplacian filter and a Mastoidal re-referencing and confirmed those described below (Perrin et al., 1989). Then, EEG signals were resampled at 128 Hz and divided into 9 s epochs corresponding to 2 s before and 7 s after the motor task for each run. Finally, we removed the trials containing muscle artifacts that may have affected ERD/ERS modulations. For this purpose, we used the EMG electrode present throughout the experiment. We also eliminated trials which included ERDs and ERS outlayers (i.e., ERDs and ERSs that significantly exceeded the confidence interval for the same run). The number of trials deleted are described in the corresponding result section (see section 3.1).

### 2.6. Time-Frequency Analysis

To analyze the differences between all four conditions, we performed an event-related spectral perturbation (ERSP) analysis between 8 and 35 Hz using EEGLAB. We used a 256 point sliding fast Fourier transform (FFT) window with a padratio of 4 and computed the mean ERSP 2s before the task to 7 s after the task. ERSP allows to visualize event-related changes in the average power spectrum relative to a baseline of 1.5 s taken 2 s before the auditory cue for C1 and C2, and 2 s before stimulation for C3 and C4 (Brunner et al., 2013). A surrogate permutation test (*p* < 0.05; 2,000 permutations) from the EEGLAB toolbox was used to validate differences in terms of time-frequency of this ERSPs.

### 2.7. Topographies

Brain topography allowed us to display the possible changes over different electrodes on the scalp in order to localize which part of the brain was involved when the subject performed the requested task. In particular, it allowed us to understand how MI + MNS and MNS conditions can be discriminated and which time parameters we can choose to guide the classification. We have decided to compute ERSPs in a merged band (mu+beta, 8–30 Hz) for MI + MNS and MNS conditions (**Figure 5**). A surrogate permutation test (*p* < 0.05; 2,000 permutations) from the EEGLAB toolbox was used to validate differences in terms of localization of this ERSPs. In addition to this analysis, we applied a false discovery rate (FDR) correction test in order to clarify how the false discovery rate was controlled for multiple comparisons. This test consists of repetitively shuffling values between conditions and recomputing the measure of interest using the shuffled data. It was performed by drawing data samples without replacement and is considered suitable to show the difference between MI + MNS and MNS conditions (Manly, 2006).

### 2.8. ERD/ERS Quantification

We compute the ERD/ERS% using the “band power method” (Pfurtscheller and Lopes da Silva, 1999).

(1) $$\begin{array}{r} ERD/ERS\%=\frac{\bar{x^{2}}-\bar{BL^{2}}}{\bar{BL^{2}}}\times 100, \end{array}$$

where $\bar{x^{2}}$ is the average of the squared signal smoothed using a 250 ms sliding window with a 100 ms shifting step, $\bar{BL^{2}}$ is the mean of a baseline segment (1.5 s) taken 2 s before the auditory cue of the corresponding trial, and ERD/ERS% is the percentage of the oscillatory power estimated for each step of the sliding window. A positive ERD/ERS% indicates a synchronization whereas a negative ERD/ERS% indicates a desynchronization. This percentage was computed separately for all EEG channels. The EEG signal was filtered in the mu rhythm (7–13 Hz), in the beta band (15–30 Hz), and in the mu+beta band (8–30 Hz) for all subjects using a 4th-order Butterworth band-pass filter.

ERD and ERS are difficult to observe from the raw EEG signal. Indeed, an EEG signal expresses the combination of activities from many neuronal sources. We used the averaging technique to represent the modulation of power of the mu and beta rhythms during MI, MNS + MI, and MNS conditions (**Figure 6**) since it is considered one of the most effective and accurate techniques used to extract events (Pfurtscheller, 2003; Quiroga and Garcia, 2003).

### 2.9. Classification

The classification was performed for the following classes: RM vs. Rest, MI vs. Rest, and MI + MNS vs. MNS. For RM and MI conditions, each trial was segmented into a motor task time for classification during the RM or the MI task and a rest time for classification during the resting state, both lasting 2.5 s. The time-window of motor task started 0.5 s after the go signal for the MI activity (1st beep), and the rest time windows started 3 s before the go signal. For MI + MNS and MNS conditions, we selected a time window of 3 s starting 0.5 s before the median nerve stimulation for all trials of both conditions. The recorded EEG signals were bandpassed using a 5th-order Butterworth filter between 8 and 30 Hz. For each classes, we collected a total of 52 trials.

We computed the performance of four different classification methods in a 4-fold cross-validation scheme. The first one uses a Linear Discriminant Analysis classifier (LDA) trained and evaluated using Common Spatial Pattern (CSP) features generated from the first and last 4 CSP filters (Blankertz et al., 2008) (referred to as CSP+LDA). The CSP method is widely used in the field of MI-base BCI, as it provides a feature projection onto a lower dimensional space that minimizes the variance of one class while maximizing the variance of the other. The other three classifiers are Riemannian Geometry based classification methods. Riemannian geometry based methods work with the covariance matrices of each trial, which live on the Riemannian manifold of symmetric positive definite matrices. These features have therefore the advantage of being immune to linear transformations (Barachant et al., 2010) First, we used the covariance matrix of each trial and applied the Minimum Distance to Riemannian Mean algorithm (MDM) to classify them, as in Barachant et al. (2010). Since this method produces a high-dimensional feature space, we trained a second instance of the MDM algorithm using a spatially filtered signal. The signal was, once more, generated using the first and the last 4 CSP filters. Finally, we computed the Riemannian barycenter of all covariance matrices in the dataset, and projected them onto the tangent space at that point. Then, since the tangent space is a Euclidean space, we trained and used a Linear Regression classifier (TS+LR). We chose to apply a paired *t*-test (two-sided) to show the significant difference about accuracy obtained for MI vs. Rest and MI + MNS vs. Rest with the TS + LR classifier (**Figure 8**, *p*-value < 0.01).

### 2.10. Software

Signal recording (EEG and EMG), synchronization/control of the median nerve stimulator and sound beep generation was designed with OpenViBE software (Renard et al., 2010). Data processing and analysis of ERD/ERS modulations were performed using MATLAB 2015b (MathWorks, Inc., Natick, MA, United States). All the classification algorithms were performed using the same computer and same software, making use of the Scikit Learn Python 2.7 machine learning package (Pedregosa et al., 2011).

## 3. Result

### 3.1. Behavioral result

Behavioral result includes two reaction times for the real movement between the auditory cues (first and second beep) and the subsequent motor task (pressing or releasing the button). It also includes the number of trial rejected because of acquisition artifacts.

#### 3.1.1. Reaction Time

For the Real Movement condition, the reaction time between the first beep and the movement start was 0.5948 s ± 0.1929. The reaction time between the second beep and the movement stop was 0.5038 s ± 0.1174. These two reaction times can be considered as normal in the light of the literature on this domain (Jain et al., 2015).

#### 3.1.2. Removing Trials

For each condition, 832 trials were acquired (52 for each subject). Due to the presence of artifacts acquired during the experiment, we used an artifact rejection script to remove the most important ones. We removed 125 (15%) trials for the RM condition, 119 trials (14,3%) for the MI condition, 114 trials (13,7%) for the MNS condition, 138 trials (16,6%) for the MI+MNS condition. The removed artifacts are homogeneously distributed among the subjects.

### 3.2. Time frequency

**Figure 4:**
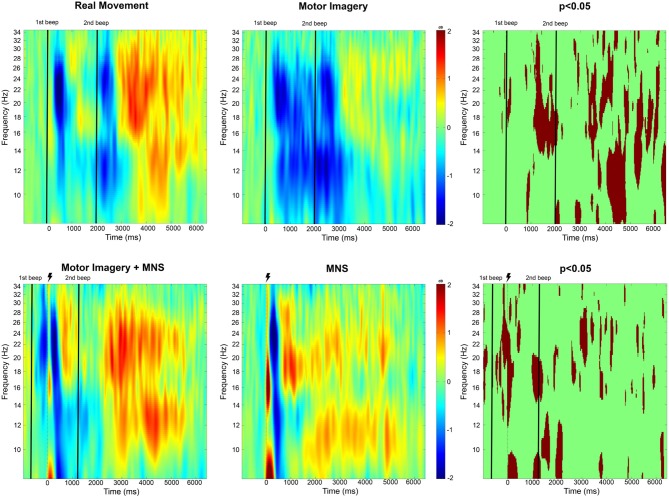
Time-frequency grand average analysis (ERSP) for Real movement, Motor Imagery, Motor Imagery + MNS, and MNS conditions for electrode *C*_3_. A black line indicates when the motor task started and finished. A flash picture indicates when the median nerve stimulation started. A red color corresponds to a strong ERS and a blue one to a strong ERD. Significant difference (*p* < 0.05) are shown in the final part of the figure.

The time-frequency maps display the signal's power evolution and are useful to establish the frequency and time windows in which ERSP appears (Figure 4).

#### 3.2.1. Real Movement and Motor Imagery

For the Real Movement condition (C1), we can observe two separate ERDs during the motor task in both mu (7–13Hz) and beta (15–30Hz) band (Figure 4). In the beta band, the first ERD starts 300 ms after the auditory cue and switches to an ERS 1 s later. The second ERD appears after the end-of-task beep and disappears 750 ms later. In the mu frequency band, instead of an ERS there is only a slight decrease of the desynchronization. A post-movement beta rebound (PMBR) arises in the beta band and shortly after in the mu band.

Throughout the Motor Imagination condition (C2), a continuous ERD occurs in both mu and beta band (Figure 4). It starts 300 ms after the auditory cue and lasts 1,200 ms after the end-of-task beep. The statistical comparison (*p* < 0.05) shows a significant difference between the MI constant ERD and the RM interrupting ERS. Additionally after the motor task in C1, there is an ERS in the mu band which doesn't exist for C2. Finally, PMBR for MI seems weaker than the rebound for RM.

#### 3.2.2. Median Nerve Stimulation During Motor Imagery

In the case of median nerve stimulation (MNS) during rest state (C3), a powerful and robust ERS appears immediately (0–250 ms) after the stimulation in low mu (7–10 Hz) and low beta (15–22 Hz) (Figure 4). For the rest of this article, this very first ERS will be named post-stimulation rebound (PSR). Then, the MNS generates an ERD (first in high beta) lasting 500 ms followed by a second rebound in both bands. MI + MNS is characterized by the presence of an pre-stimulation ERD. Interestingly, the PSR is almost nonexistent in this condition (*p* < 0.05) but the ERD (250–500 ms after the MNS) is very similar. The MNS-generated beta rebound appears less powerful than the one from C3 and, instead of a return to baseline, a continuous mu ERD last until the end of the motor task. Finally a third rebound appears in both frequency bands 1,200 ms after the motor task.

### 3.3. Topographic Map

**Figure 5:**
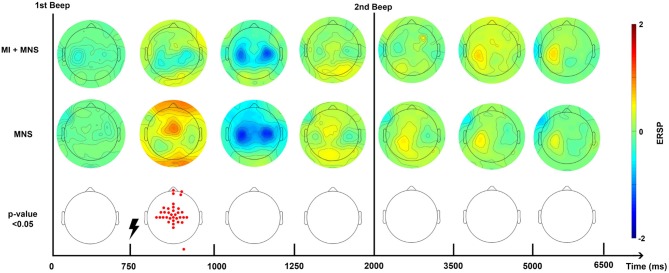
Topographic map of ERD/ERS% (grand average, *n* = 16) in the alpha/mu+beta band during two conditions: MI + MNS and MNS only. A red color corresponds to a strong ERS and a blue one to a strong ERD. A black line indicates when the motor imagery started or finished for the MI + MNS condition. Red electrodes indicate a significant difference between the two conditions (*p* < 0.05).

Analysis of these time-frequencies maps showed that both mu (7–13 Hz) and beta (15–30 Hz) bands were impacted in term of synchronization/desynchronization in all four conditions. Since the previous results for C1 and C2 are consistent with the literature and the purpose of this study is to discriminate C3 and C4, we will only look into the last two conditions. Consequently, a larger frequency band (8–30Hz) was chosen to analyse the ERD and ERS localization. Figure 5 shows that the MNS doesn't have the same impact depending of the subject being in a rest or MI state. Indeed, 250 ms after the MNS there is a significant difference on several electrodes in term of PSR (mostly on motor, pre-motor, and sensorimotor areas both central and bilateral). A bilateral ERD appears for both condition 500 ms after the MNS followed by a beta rebound slightly diminished for the MI + MNS condition than for MNS only.

According to this Figure 5, and in the views of discriminating these two conditions, we distinguished a promising time window which should start just before the MNS and stop after the end-of-task beep. For the MI + MNS condition, this time window includes the (i) pre-stimulation MI-generated ERD, (ii) the abolished PSR, and (iii) the diminished MNS-generated beta rebound.

### 3.4. ERD and ERS modulation

**Figure 6:**
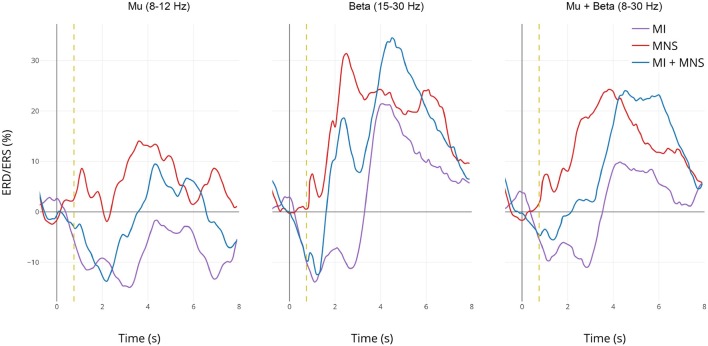
Grand average (*n* = 16) ERD/ERS% curves in the mu (7–13 Hz), the beta (15–30 Hz), and the mu+beta (8–30 Hz) bands for MI (in violet), MI + MNS (in blue), and MNS (in red) conditions for electrode *C*_3_. The yellow bar at 750 ms corresponds to the median nerve stimulation performed. For the MI and MI + MNS conditions, the MI starts at 0 s and ends at 2 s.

In accordance with the results obtained from the time-frequency and topographic analyses, the ERD and ERS modulations have been computed for three frequency bands, mu: 8–12 Hz, beta: 15–30 Hz and mu+beta band: 8–30 Hz for all subjects. The Figure 6 represents the grand average of all subjects for the C3 electrode.

#### 3.4.1. Mu band

In the mu frequency band, a desynchronization appears and reaches –15% during both MI task (C2 in purple and C4 in blue during 0–2,000 ms). This observation confirms that the ERD created by the MI task isn't impacted by the following MNS (besides the slight PSR in C4) in the mu band. Logically, this desynchronization doesn't exist for the MNS condition. After the motor task, a slight rebound appears for MNS and MI + MNS condition.

#### 3.4.2. Beta Band

The ERD in the beta frequency band behaves similarly to the ERD in the mu band, only C2 and C4 display this desynchronization. However, during MI + MNS, the ERD is shorter (1,700 ms) than the one in MI only. Logically, during C3 no ERD appears. As seen on the topography and time-frequency figures, an ERS appears for all conditions 3 and 4 after the stimulation. This ERS is partially diminished for MI + MNS but is followed by a stronger post-motor task rebound (33% at 4,500 ms), also present for C2.

#### 3.4.3. Mu+Beta Band

If we merge the two frequency bands, the behavior of ERD and ERS is particularly interesting since the difference between condition 3 and 4 is strong on a 0–3,000 ms time window. On the same note, after 3,000 ms, the condition 3 ERS starts to disappear but the MI + MNS ERS keeps a level of 24%. Those results highlight the interest of the 8-30Hz frequency band if we seek to discriminate C3 and C4.

### 3.5. Classification

In order to verify that a MNS is useful as a trigger to detect a movement intention, we decided to compare the classification score obtained for the traditional MI vs Rest class and our MI + MNS vs. MNS class.

**Figure 7:**
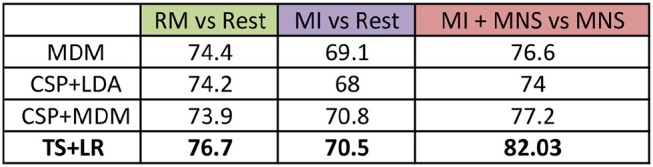
Grand average accuracies obtained by 4 differents classifiers (MDM, CSP + LDA, CSP + MDM, TS + LR) for the 3 conditions (RM, MI and MI + MNS) in the mu + beta band (8–30 Hz).

We pre processed our data in the following manner: (a) the frequency band is restricted to 8–30Hz; (b) we consider only the premotor frontocentral, primary motor cortex, and somatosensorial central and occipital electrodes; and (c) the classification time window is [–0.5 to 2.5 s] for MI+MNS vs. MNS, [0.5–3 s] for MI vs. Rest. These values are based on the existing literature for MI-based BCI. The average classification accuracies between a MI and a rest period, and between MI + MNS and MNS were computed for 4 different classifiers (MDRM, CSP+LDA, FgMDRM, TS+LR, see Figure 7). TS+LR gave the best results for both classifications, which was not an unexpected result. Indeed, this classification method combines the invariance properties of Riemannian Geometry-based methods and the well-established linear regression method. For the rest of the results, we only use the output of the TS+LR classification method.

**Figure 8:**
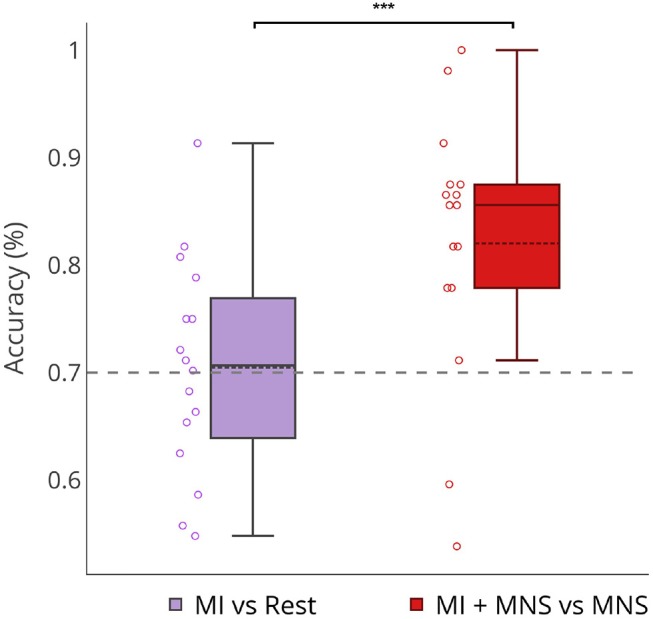
Boxplots showing the distribution of average classification accuracies (*n* = 16) for MI vs Rest and MI + MNS vs. MNS class. ^***^p-value < 0.001.

Figure 8 shows that a MI + MNS vs. MNS classification allows better accuracies than a MI vs. Rest classification, and proves that a MNS can be used as a trigger and improves MI detection.

**Figure 9:**
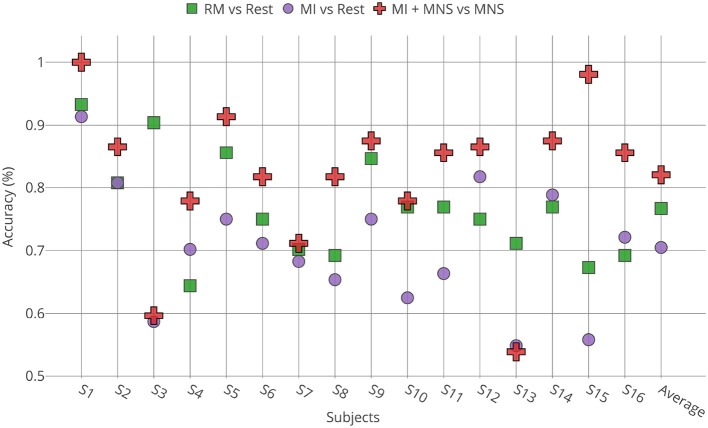
Accuracies obtained for all subjects (*n* = 16) by TS + LR analyses in the 8–30 Hz for the 3 conditions (RM, MI and MI + MNS).

Individual classification shows a greater classification performance with MNS for 14 subjects (Figure 9). Only subject 3 and subject 13 shows better performance for a MI vs. Rest classification, but the results don't exceed 60%.

## Discussion

This work confirmed that median nerve stimulation indeed generates ERD and ERS in the motor cortex. When the median nerve is stimulated during an intention of movement, those ERD and ERS are significantly impacted. Based on these differences, we confirmed that a classifier is able to discriminate a stimulation during a rest state from a stimulation during an intention of movement. Our results show that the TS+LR classifier performs better for the two conditions involving a MNS, in comparison to the typical discrimination task between rest state and MI state. This confirms the feasibility of implementing a MNS-based BCI to detect intraoperative awareness. In this section we discuss the consistency of the ERD and ERS modulation for all our conditions, including the MNS impact on MI, the benefits of our classifier and how our work could be used in the intraoperative awareness situation.

###  ERD/ERS Modulations During a Real Movement and a Motor Imagery

According to Erbil's work (Erbil and Ungan, 2007), maintaining a real movement creates an ERS. This would explain the results obtained in Figure 4, showing two distinct ERDs separated by an ERS during the real movement task. During the MI task, a continuous ERD is observed (Figures 4, 6) which suggests that the subjects applied the instruction of maintaining the MI during 2 s. The continuous ERD and the weaker post-MI ERS, in comparison with the post-RM ERS, are consistent with the findings of several articles (Pfurtscheller and Neuper, 1997, 2001; Neuper and Pfurtscheller, 2001; Filgueiras et al., 2017). In addition, a previous study showed that a closed-eyed condition generated a stronger ERD in the mu band (Rimbert et al., 2018).

###  ERD/ERS Modulations During a Median Nerve Stimulation

Our results showed that MNS modulates the ERD and ERS in the EEG signal from the motor cortex. More precisely, MNS produces a first PSR (Figure 4) which is visible in all subjects (not presented in this article). This PSR was not mentioned in the very few articles that discussed this topic (Salenius et al., 1997; Neuper and Pfurtscheller, 2001) and could be interpreted as an attention marker (Saleh et al., 2010). Five hundred milliseconds after the MNS, a strong ERD appears in the mu and beta band and had already been mentioned by Salenius in the beta band for MEG (Salenius et al., 1997) and by Neuper in both bands for EEG (Neuper and Pfurtscheller, 2001).

###  Impact of a Median Nerve Stimulation During a MI

Several articles have already shown that performing a MI during median nerve stimulation has an impact on motor patterns previously generated by MNS (Salenius et al., 1997; Schnitzler et al., 1997; Neuper and Pfurtscheller, 2001). Our results confirm that the intention to move tends to modify the ERD/ERS normally present during a single MNS. Indeed, the PSR is almost abolished during the MI (Figure 4). In contrast, the post-stimulation ERD is unchanged while the second ERS tends to be decreased as already shown in the literature (Neuper and Pfurtscheller, 2001). Interestingly, our results indicate that the mu band (500–1,400 ms) is unaffected by the MNS effect, which suggests a functional difference between the two frequency bands. Finally, the post MI rebound is stronger than in MI condition alone, which implies a rebound additive phenomenon.

###  Median Nerve Stimulation As an Innovative Trigger for Intraoperative Awareness Detection

Intraoperative awareness is an uncertain phenomenon. There is no absolute way to predict when it will occur (Pandit et al., 2014). However, several studies have shown that moving is a patient's first reflex to warn about his awakening (Ghoneim et al., 2009). Theoretically, if a BCI could detect a patient's intention of movement during his awakening, it would need to use classification without any trigger, since it's impossible to know the moment when the patient tries to move. While there exist some BCIs that do not use time markers or triggers (known as asynchronous BCIs), the literature clearly shows that their classification rate is lower than that of a synchronous BCI with triggers (Nicolas-Alonso and Gomez-Gil, 2012).

Our results show a performance of 70% for MI vs Rest classification with a trigger. In the absence of this trigger these results would be weaker (Figure 8). On the other hand, our MI+MNS vs. MNS classification displays accuracy results of 80%. This method brings about the possibility of a more efficient way to detect intraoperative awareness.

According to our results, we can imagine a routine system where the patient would be stimulated at the median nerve (e.g., every 5 s), while a passive BCI device would analyze the ERD and ERS modulations of the motor cortex to see if the patient intends to move or not. In case of such BCI could detect a modulation suggesting an intention of movement, the anesthesiologist could therefore adjust the doses of anesthetics.

###  Perspectives

#### Getting Closer to the Anesthetized State

Our study was conducted on non-anesthetized subjects, and as shown by Blokland et al., we can expect some difference in the cerebral activity behavior once propofol is used (Blokland et al., 2016). Our results will be confirmed during a clinical protocol where the same conditions will be used on voluntary anesthetized subject. If we can find similar results on anesthetized subject, we also plan to repeat the experimentation on subjects with induced neuromusclar blockade in order to study real movement intention instead of motor imagination. A final experiment we could combine both condition with paralyzed and anesthetized patient in order to investigate if the combination could change the results.

#### Getting Closer to the Implementation

Another perspective we are interested in is to create a new way to classify our data online. We need to have an easy-to-implement classification pipeline in order to make this hypothetical device as practical to use as possible. One of the most important parts of a BCI pipeline is the calibration of the pre-processing and classification parameters. It is clear that in this application, calibration data from the same user can be difficult to obtain. A thorough analysis of existing datasets, such as leave-one-subject-out analyses could enable us to determine pre-processing parameters, including the optimal frequency bands or the number of electrodes required to obtain good results.

**Figure 10:**
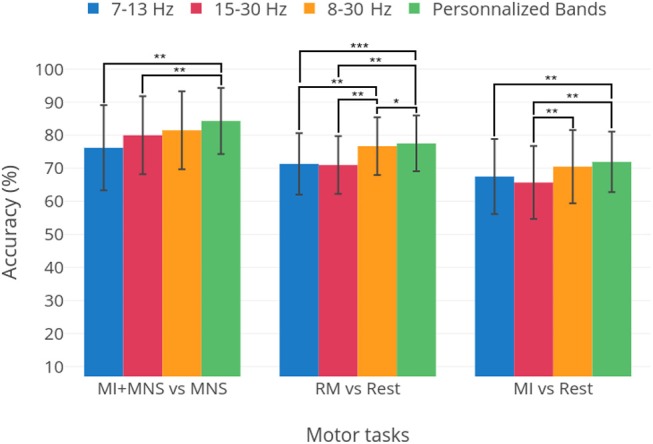
Average performances and standard deviation for thee classification tasks: MI+MNS vs. MNS, RM vs. Rest, and MI vs. Rest. The three first bars show the results obtained for the 7–130, 15–30, and 8–30 Hz frequency bands. The fourth bar labeled “Personalized bands” is the average and standard deviation of results when the best frequency band for each subject is chosen, i.e., the frequency band yielding the highest performance. Statistical significances are displayed as well, obtained in a student's *t*-test. The classifier is the TS+LR, described in section 2.9. ^*^p-value < 0.05, ^**^p-value < 0.01, ^***^p-value < 0.001.

A preliminary analysis, presented in Figure 10 shows the optimal results for each patient for three frequency bands: μ, β and 8–30 Hz. These results are compared to selecting a single frequency band for all subjects. We see that, although for a fixed frequency band selection, the 8–30 Hz range is the apparent best choice, it is clear that personalizing the choice of a frequency band yields better results. This warrants the use of methods that improve classification accuracy by adapting the classification pipeline to each subject (Ang et al., 2012; Duprès et al., 2016). Nevertheless, our results indicate that the difference is not significant for the MI vs Rest and MI+MNS vs MNS classifications (*p* < 0.05). Moreover, the implementation of methods that depend on data coming from the same BCI session might be hard to implement in clinical settings.

In future works, we intend to address such issue by using transfer learning approaches such as Riemannian geometry based methods (Rodrigues et al., 2018) or optimal transport based methods (Gayraud et al., 2017). Indeed, transfer learning has proven to be very effective in designing BCIs with little or no calibration for a new user (Lotte, 2015).

Finally, one last thing we wish to study is the impact of MNS at various times during a MI task. In this study we stimulated our subjects at the same time for the entire experimentation (750 ms after the MI task start), but in a real surgery, the MNS would intervene at different times and the cerebral activity could be modulated differently.

## Conclusion

In this study, we verified that median nerve stimulation modulates the motor cortex by first generating an ERD during stimulation and then an ERS post-stimulation. In addition, we discovered a new Post-Stimulation Rebound ERS which appears 250 ms after the stimulation in the mu and low beta band. Median nerve stimulation combined with the intention to move, i.e., the MI, has a significant impact on the ERD and ERS generated by the MNS. Indeed, despite the fact that the ERD was unaltered, the PSR is almost abolished and the rebound in the beta band is diminished. Those differences have resulted into a high accuracy classification. With these findings, we show that a BCI based on MNS is more effective than a BCI based on a MI state vs. rest. This innovative approach may improve the detection of intraoperative awareness during general anesthesia.

## Ethics Statement

This study was carried out in accordance with the recommendations of COERLE ethic committee of INRIA with written informed consent from all subjects. All subjects gave written informed consent in accordance with the Declaration of Helsinki. The protocol was approved by the COERLE ethic committee.

## Author Contributions

SR and PR conceived, designed, and performed the experiments. SR, PR, NG, DS, and LB analyzed the data, contributed reagents, materials, analysis tools, prepared figures and/or tables, authored or reviewed drafts of the paper, approved the final draft.

### Conflict of Interest Statement

The authors declare that the research was conducted in the absence of any commercial or financial relationships that could be construed as a potential conflict of interest.
